# Structural basis for the structural dynamics of human mitochondrial chaperonin mHsp60

**DOI:** 10.1038/s41598-021-94236-y

**Published:** 2021-07-20

**Authors:** Joseph Che-Yen Wang, Lingling Chen

**Affiliations:** 1 Department of Microbiology and Immunology, The Pennsylvania State University College of Medicine, 500 University Drive, Hershey, PA 17033 USA; 2 Department of Molecular and Cellular Biochemistry, 212 S. Hawthorne Dr., Bloomington, IN 47405 USA

**Keywords:** Cryoelectron microscopy, Chaperones

## Abstract

Human mitochondrial chaperonin mHsp60 is essential for mitochondrial function by assisting folding of mitochondrial proteins. Unlike the double-ring bacterial GroEL, mHsp60 exists as a heptameric ring that is unstable and dissociates to subunits. The structural dynamics has been implicated for a unique mechanism of mHsp60. We purified active heptameric mHsp60, and determined a cryo-EM structure of mHsp60 heptamer at 3.4 Å. Of the three domains, the equatorial domains contribute most to the inter-subunit interactions, which include a four-stranded β sheet. Our structural comparison with GroEL shows that mHsp60 contains several unique sequences that directly decrease the sidechain interactions around the β sheet and indirectly shorten β strands by disengaging the backbones of the flanking residues from hydrogen bonding in the β strand conformation. The decreased inter-subunit interactions result in a small inter-subunit interface in mHsp60 compared to GroEL, providing a structural basis for the dynamics of mHsp60 subunit association. Importantly, the unique sequences are conserved among higher eukaryotic mitochondrial chaperonins, suggesting the importance of structural dynamics for eukaryotic chaperonins. Our structural comparison with the single-ring mHsp60-mHsp10 shows that upon mHsp10 binding the shortened inter-subunit β sheet is restored and the overall inter-subunit interface of mHsp60 increases drastically. Our structural basis for the mHsp10 induced stabilization of mHsp60 subunit interaction is consistent with the literature that mHsp10 stabilizes mHsp60 quaternary structure. Together, our studies provide structural bases for structural dynamics of the mHsp60 heptamer and for the stabilizing effect of mHsp10 on mHsp60 subunit association.

## Introduction

Human mitochondrial mHsp60 is essential for mitochondria as it is required for mitochondrial protein transport, folding and assembly^1–4^. Due to its roles in human health, mHsp60 has been proposed as potential biomarkers and drug targets^5–8^. mHsp60 belongs to an essential protein family called chaperonin that are conserved among the three kingdoms of life^9^. The main function of chaperonins is to mediate folding of cellular proteins. Detailed molecular mechanism of chaperonins has been extensively investigated using the *E. coli* chaperonin GroEL and its cochaperonin GroES^10–14^. GroEL consists of 14 subunits assembled in two heptameric rings forming two central cavities, and each subunit consists of three domains, apical, intermediate and equatorial domains. Proteins in non-native conformations bind to the central cavity, and in an ATP-dependent mechanism they are sequestered into the cavity upon GroES binding to the end of the substrate-loaded cis heptameric ring. The enclosed GroEL-GroES chamber provides the substrate protein an isolated environment favorable for folding. Binding of ATP to a second GroEL ring, trans to the substrate-loaded GroEL-GroES chamber, trigger dissociation of the GroEL-GroES folding chamber, releasing the substrate protein. In support of the essential role of the second ring in GroEL, a single-ring GroEL, GroEL^SR^, where conserved inter-ring contacts including a conserved salt bridge are disrupted, is nonfunctional with GroES in vivo^15^, because the GroEL^SR^-GroES complex does not dissociate^16^.

Unlike GroEL, mHsp60 exists as single ring^17^, and mHsp60 lacks the two pairs of salt bridge found in the inter-ring interface in the double-ring GroEL^18^, suggesting that mHsp60 and its cochaperonin mHsp10 may function in a single-ring mechanism other than the double-ring mechanism of GroEL-GroES. In support of the single-ring mechanism, mHsp60 interacts transiently with mHsp10^19^, thus the dissociation of the mHsp60-mHsp10 chamber does not require the action from a second ring. However, an earlier model^20–23^, supported by the crystal structure of an inactive mHsp60 variant^24^ complexed with mHsp10 in a double-ring football-shaped conformation^25^, assumes that mHsp60 functions via a GroEL-like double-ring mechanism. In the recent revised model, the single-ring mechanism has been incorporated in addition to the double-ring mechanism^26^. In the studies, cryo-EM shows that mHsp60 forms both the single- and double-ring mHsp60-mHsp10 complexes in the presence of ADP. Structure-based mHsp60 variants of single-ring only and covalent bonded double-ring are shown active in folding of a substrate protein and in substituting GroEL-GroES to support *E. coli* cell growth. Thus, it is proposed that mHsp60-mHsp10 can function as either single- or double-ring mechanisms based on the specific mitochondrial substrates.

Detailed mechanistic investigations on mHsp60-mHsp10 had been hindered by difficulties in preparing mHsp60 until recently. Purified mHsp60 dissociates readily to nonfunctional monomers^17,27^, and mHsp60 has been purified as a double-ring conformation that when in the presence of mHsp10 cannot hydrolyze ATP without the substrate protein^21^, highlighting the difficulties in mHsp60 preparation. In this report, we purified active mHsp60 in single-ring conformation, and determined mHsp60 structure using cryo-EM. We found that mHsp60 contains several unique residues that decease inter-subunit sidechain interactions and shorten the inter-subunit β sheet, resulting in reduced inter-subunit interface when compared with GroEL. These residues are conserved among higher eukaryotic mitochondrial chaperonins. Our structural analysis with the reported single-ring mHsp60-mHsp10 shows that the mHsp60 subunit interface is increased as a result of mHsp10 binding. Note that a cryo-EM structure of mHsp60^28^ was reported during our manuscript submission, however, without structural analysis on inter-subunit interactions and discussion on their roles on the structural dynamics of mHsp60.

## Results

### Purified mHsp60 is single ring and active

**Figure 1:**
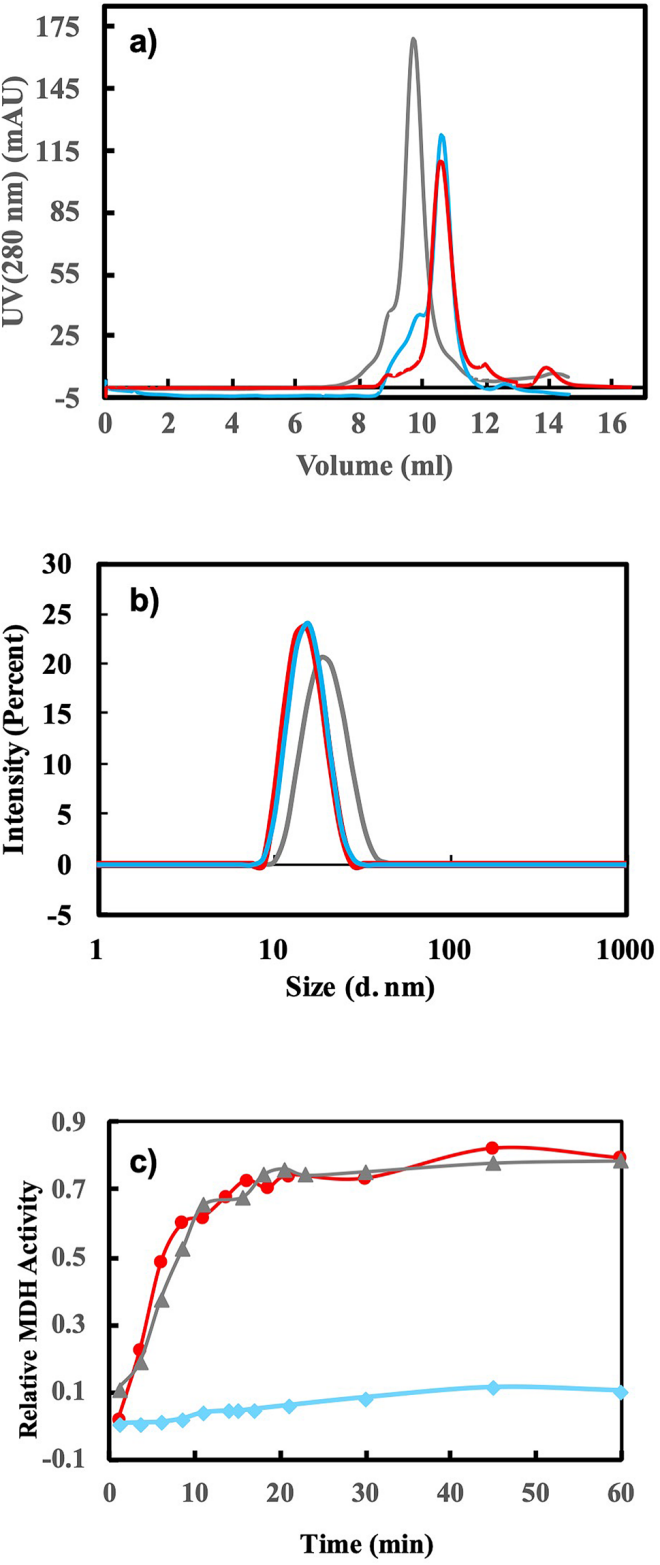
Characterizations of mHsp60. (**a**) Size exclusion chromatographic profiles, (**b**) dynamic light scattering analysis, (**c**) refolding malate dehydrogenase (MDH) of double-ring GroEL (grey), single-ring GroEL^SR^ (cyan) and mHsp60 (red). The results are summarized in Table 1.

To examine conformation of our purified mHsp60, we used size-exclusion chromatography (SEC) and dynamic light scattering (DLS) to compare mHsp60 with the double-ring GroEL and single-ring GroEL^SR^. As shown in Fig. 1a and Table 1, mHsp60 eluted at the same volume as that of GroEL^SR^, but later than that of the double-ring GroEL. Also, mHsp60 existed as a particle with a size similar to that of GroEL^SR^, but smaller than that of GroEL (Fig. 1b and Table 1). All three samples were monodisperse since their Polydispersity Index (PDI) were < 0.1. Thus, our purified mHsp60 was homogenously in a single-ring conformation, which was further supported by negative stained TEM image (Fig. S1).

**Table 1:** The molecular properties of mHsp60, GroEL^SR^ and GroEL.

Protein	Elution volume (ml)^a^	Z-Average size (d. nm)	ATPase (min^−1^)^c^	MDH yield^d^
mHsp60	10.62	14.85 (0.09)^b^	0.099 ± 0.012 0.052 ± 0.012 (w/mHsp10)	77.5% ± 2.0%
GroEL^SR^	10.62	14.62 (0.08)	0.456 ± 0.020	10.3% ± 2.1%
GroEL	9.53	18.69 (0.07)	0.422 ± 0.020	77.8% ± 1.9%

^a^SEC was carried out using a Superdex 200 10/300 GL column (GE Healthcare Life Sciences).

^b^Values in parentheses refer to Polydispersity Index (PDI).

^c^ATPase activity is normalized to the monomer concentration.

^d^MDH yield was at 60 min after the unfolded MDH was added to the chaperonin systems, and was normalized to native MDH. Without the chaperonin system, the unfolded MDH did not refold (< 5%).

We next examined the biochemical activities of the purified mHsp60. mHsp60 hydrolyzed ATP at a rate ~ 25% of GroEL (Table 1), consistent with the previous reports^19,20,29^. The intrinsic ATPase rate of mHsp60 was decreased by ~ 50% in the presence of mHsp10: the cochaperonin inhibition on the ATPase of the chaperonin is a property found in the chaperonin systems. Moreover, mHsp60-mHsp10 was as effective as GroEL-GroES in mediating folding of malate dehydrogenase (MDH) (Fig. 1c and Table 1). Thus, our purified mHsp60 was biochemically active.

### Cryo-EM analysis of mHsp60

**Figure 2:**
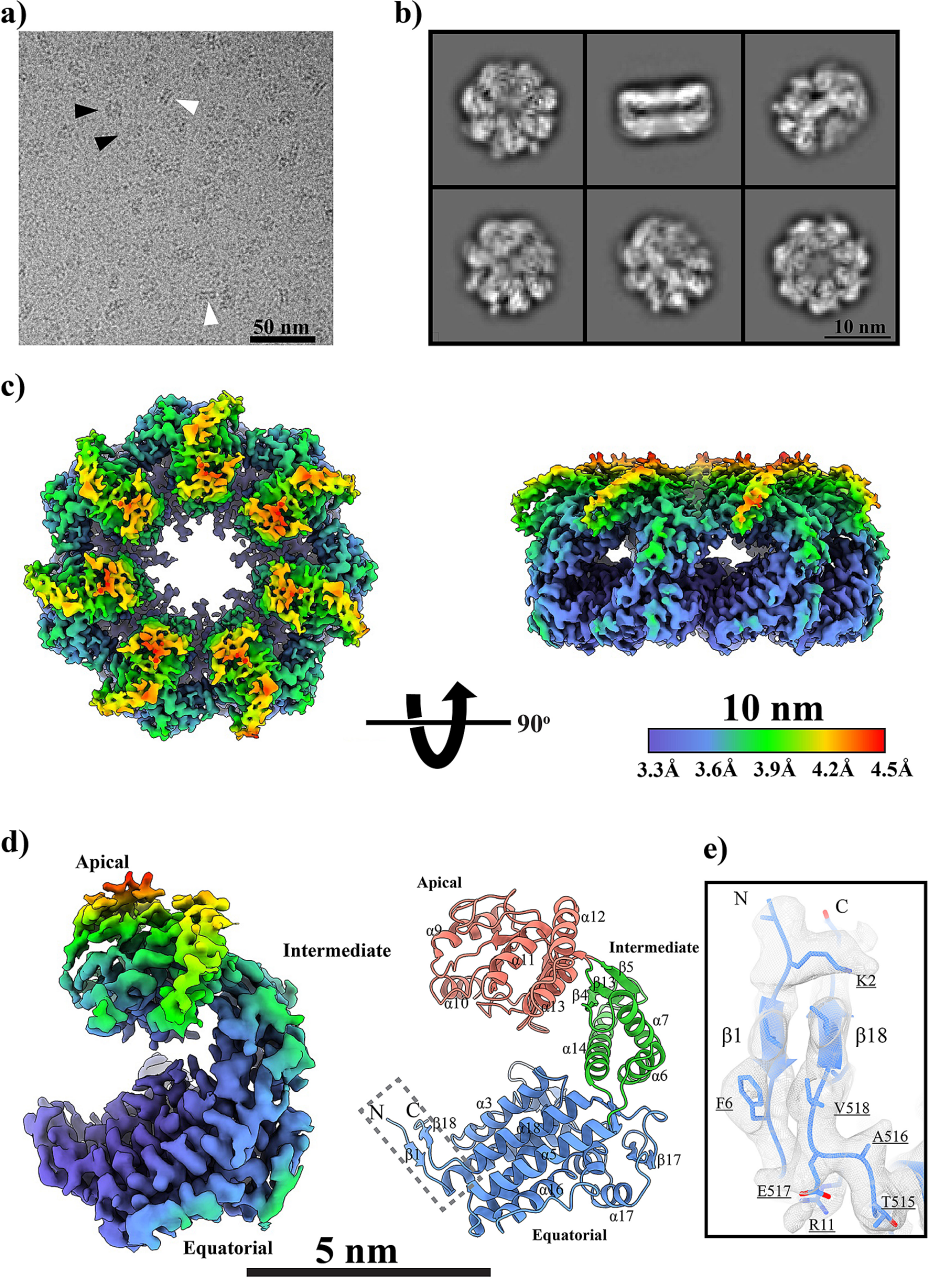
Cryo-EM analysis of mHsp60. (**a**) A representative cryo-EM micrograph. Side and tilt views are highlighted by white and black arrowheads. (**b**) Representative reference-free 2D class averages images. (**c**) Cryo-EM reconstruction of the mHsp60 heptamer colored by local resolution. (**d**) A mHsp60 subunit displayed in cryo-EM reconstruction with local resolution and in ribbon representation. Ribbon color scheme, blue, green and red for the equatorial, intermediate and apical domains, is the same as Fig. 3C. (**e**) The EM density at the region involved in the inter-subunit interaction.

Cryo-EM micrographs also confirm the single-ring conformation of mHsp60 (Fig. 2a). Top and bottom views of the 2D class averages shows seven-fold symmetry characteristics (Fig. 2b), confirming the heptameric arrangement of the mHsp60 subunits. The two stripes in the side view correspond to the apical and equatorial domains, indicating a single-ring conformation. Thus, EM studies validated our purified mHsp60 as a single-ring heptamer. The final cryo-EM map was estimated to 3.4 Å (Table 2, Fig. S2) with various local resolutions (Fig. 2c). The map (Fig. 2d) shows that the equatorial domain has the highest resolution (3.3 Å), while the apical domain has the lower resolution range (3.9–4.5 Å). Since high resolution maps reflect a homogenous ensemble of conformational states, our observations indicate that the mHsp60 equatorial domains are structurally more homogenous and better ordered than the apical domains, in agreement with crystal structural analysis of GroEL^30,31^. The homogenous and well-ordered structure of the equatorial domains is attributed by their extensive inter-subunit interactions; they contribute ~ 75% of the total inter-subunit interface in the heptamer. The regional map in the equatorial domain that is involved in the inter-subunit interactions (below) is well resolved with clear sidechain information (Fig. 2e).

**Table 2:** Cryo-EM data collection, refinement and validation statistics**.**

Reconstruction data	mHsp60 (PSU COM)	mHsp60 (NCCAT)
**Data collection information**		
Electron microscope	TFS Titan Krios	TFS Titan Krios
Operation voltage (kV)	300	300
Electron detector	Gatan K3	Gatan K3
Energy filter (slit width in eV)	30	no
Data collection software	TFS EPU	Leginon
Data collection mode	counting	Super-resolution counting
Nominal magnification	120,000 ×	81,000 ×
Pixel size in Å (super resolution)	0.84	1.069 (0.535)
Total accumulated dose (e^−^/Å^2^)	44	50–57
**Data process statistics**		
Box size (pixel)	320	240
Number of particles	196,060	182,600
CTF estimation	Gctf	CTFFIND4
Data processing software	RELION 3.1	RELION 3.1
Symmetry imposition	C7	C7
B-factor applied (Å^2^)	− 151	− 191
Final resolution (Å)	3.5	3.5
Combined B-factor (Å^2^)	− 180	
Combined resolution (Å)	3.4	
EMDB code	EMD-23217	
**Model refinement statistics**		
CC_masked_ (Model vs Data)	0.73	
CC_volume_ (Model vs Data)	0.71	
RMSD bond length (Å)	0.007	
RMSD bond angles (°)	0.699	
Ramachandran plot		
Favored (%)	88.85	
Allowed (%)	10.77	
Outliers (%)	0.38	
Clash score	12.51	
PDB code	7L7S	

### Overall structure of mHsp60

**Figure 3:**
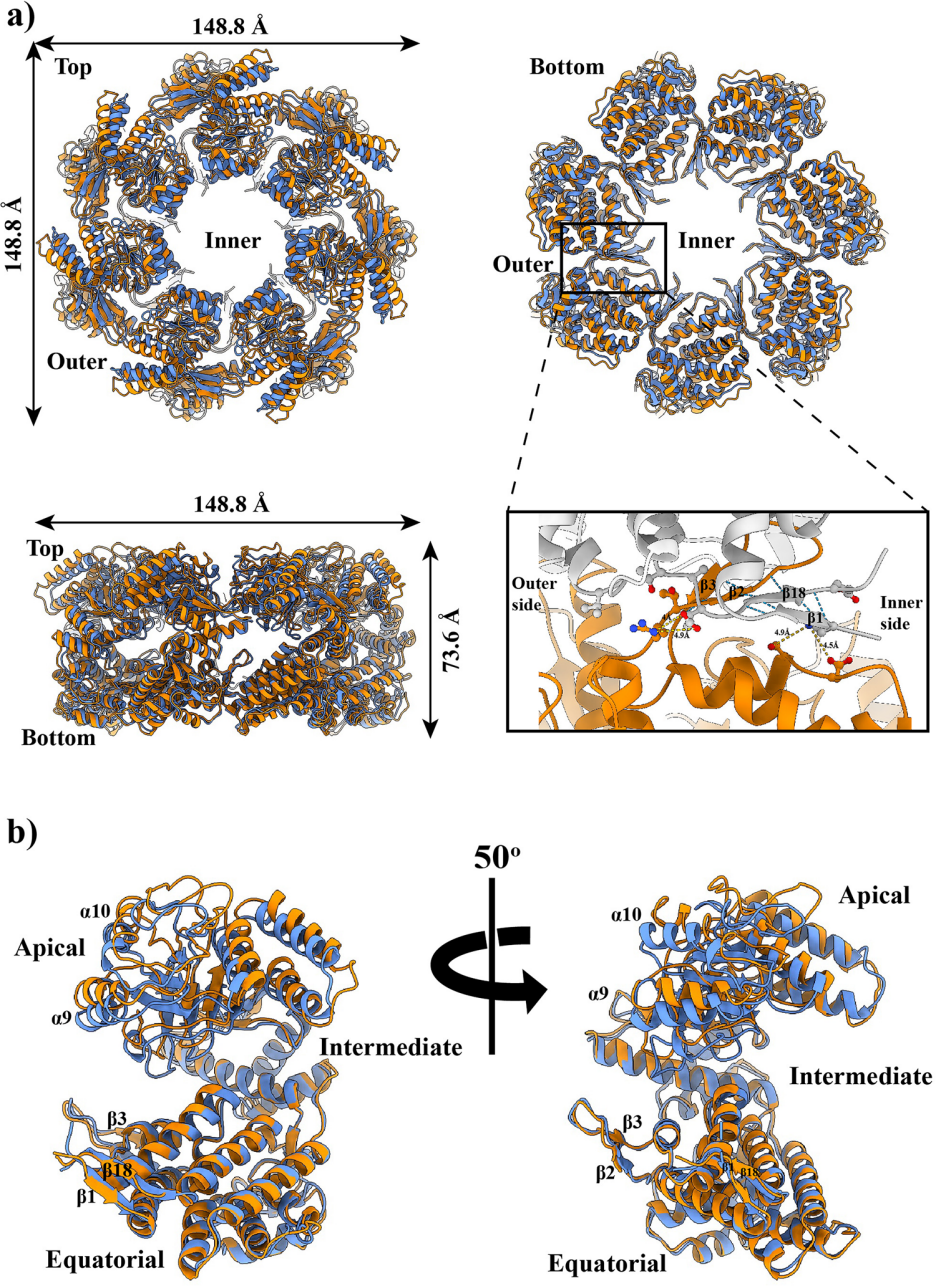

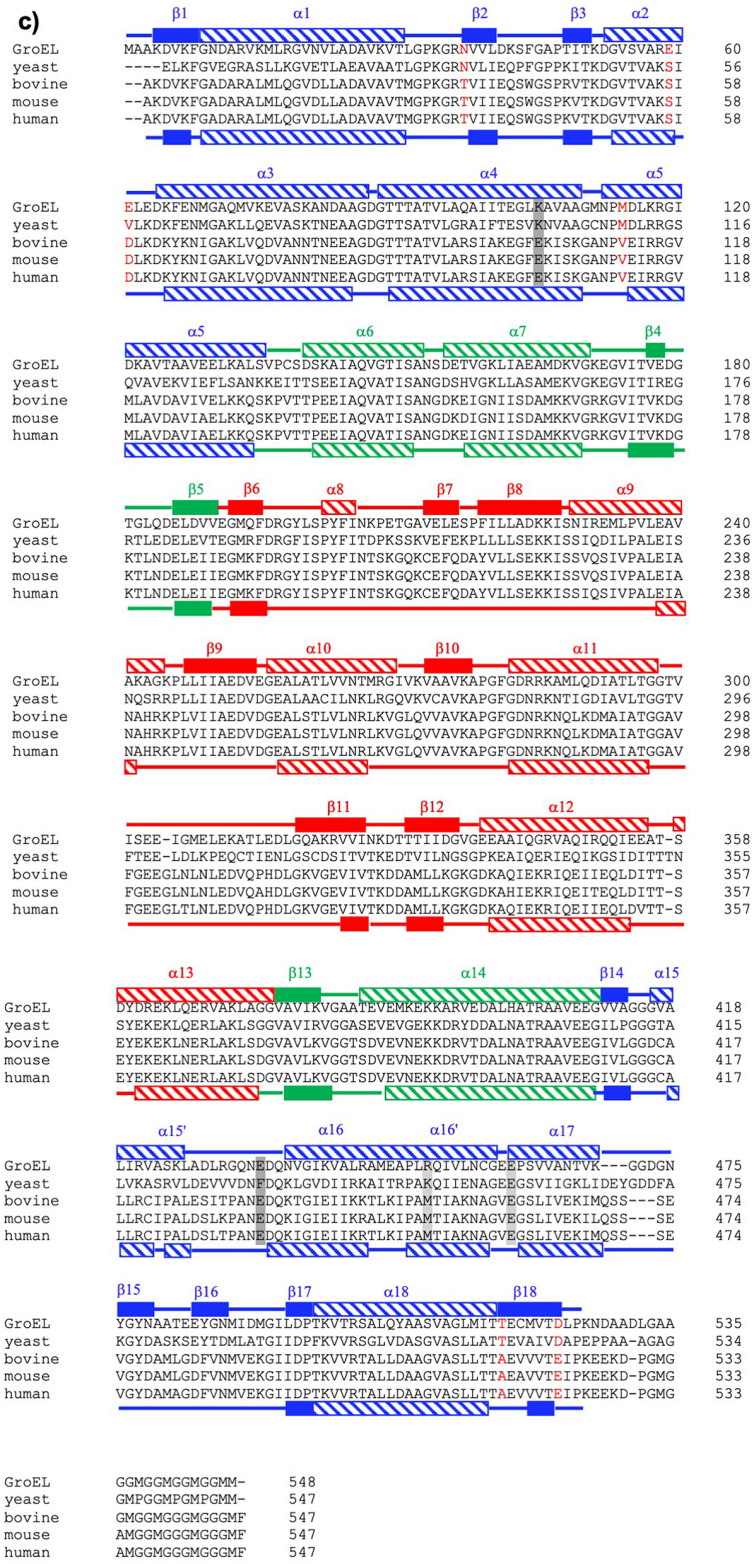

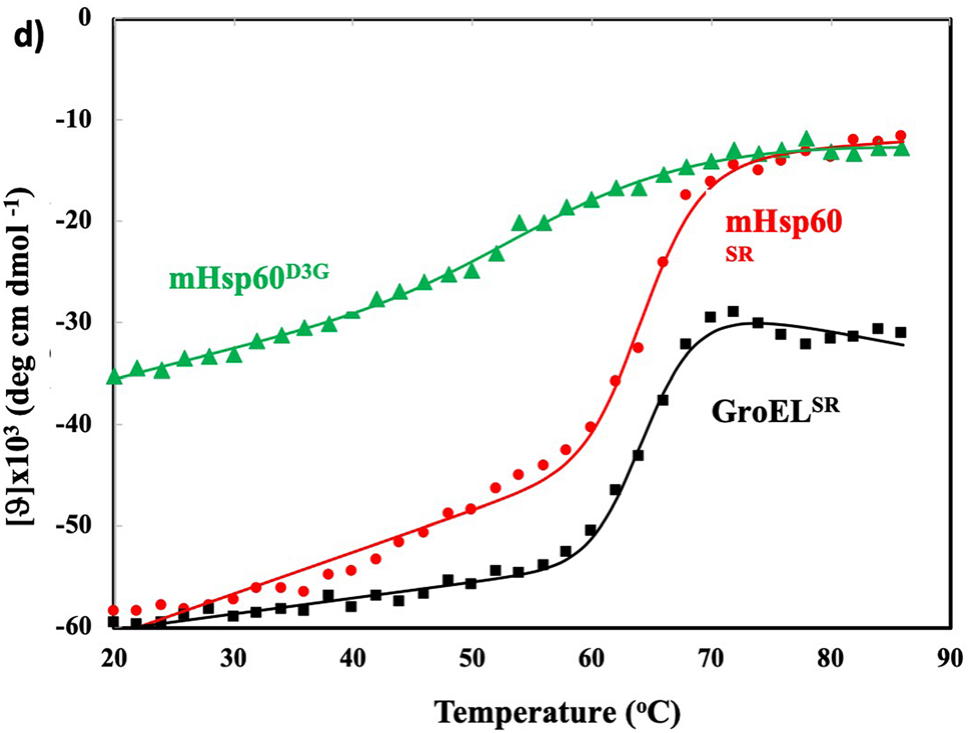
An expanded mHsp60 heptameric ring. (**a**) Superimpose of mHsp60 (orange) and a GroEL ring (blue; PDB: 5w0s) in top, bottom and side views. A four-stranded β-sheet, consisting of two strands β1 and β18 from one subunit (grey) and two strands β2 and β3 from the other subunit (orange), is located at the center of the cross-ring inter-subunit interface. Residues involved in inter-subunit interactions are highlighted in Fig. 4, and the atomic distances are summarized in Table 3. The mHsp60 heptamer and one GroEL heptamer were superimposed. (**b**) Overlay of a mHsp60 subunit with a GroEL subunit. The two equatorial domains were superimposed. (**c**) Sequence alignment among chaperonins from various sources. Secondary structures of mHsp60 and GroEL are based on Chimera: stripped rectangles for α helices and solid rectangles for β strands. Color scheme is the same as Fig. 2d. Residues involved in the inter-subunit interactions that are unique to human or higher eukaryote mouse and bovine (although yeast also has Ser at the corresponding S57 in human) are in red. Four residues forming the conserved inter-ring salt bridges are in dark (K105-E435) and light (R453-E462) shades, in GroEL numbering. For convenience, numbering of GroEL secondary structures is used here. (**d**) Thermal denaturation of single-ring chaperonins. The molar ellipticity (ϑ) was measured at 222 nm. The solid lines are non-linear regression analysis (Eq. 1). The derived ΔH_vh_, ΔG_unfolding_ and T_m_ values are listed in Table 4.

The mHsp60 heptameric ring is larger than a GroEL ring. The diameter of the mHsp60 ring is 148.8 Å, 8 Å wider than that of the GroEL ring (PDB: 5w0s; 3.5 Å cryoEM structure)—the diameter of the chaperonin ring is determined by the equatorial domains. Since the apical domains contribute to the height of the chaperonin ring, their lower resolution regional map in mHsp60 may not estimate the height of the mHsp60 ring (74.4 Å) accurately compared to that of the GroEL ring (69.1 Å); however, mHsp60 appears taller than the GroEL ring (Fig. 3a). A larger mHsp60 ring is also evident by comparing cryo-EM reconstructions (Fig. S3) of mHsp60 and GroEL (EMDB: 8750). The larger size of the mHsp60 ring suggests that the seven mHsp60 subunits may not interact as strongly as the GroEL subunits in the GroEL ring.

Between mHsp60 and GroEL, the three domains, apical, intermediate and equatorial domains, are arranged similarly but differ in their conformations. While conformations of the equatorial domains overlay well, those of the apical domains deviate (Fig. 3b). Overall, mHsp60 consists of shorter α helices and β strands than GroEL (Fig. 3c). 292 mHsp60 residues while 392 GroEL residues are involved in forming either α helices or β strands (Fig. 3c). The lower secondary structure content of mHsp60 suggests that the mHsp60 subunits are less well folded than the GroEL subunits.

### Reduced inter-subunit interactions in mHsp60

The equatorial domains contribute most of the inter-subunit interactions, accounting for ~ 75% of the total inter-subunit interface and 44 residues involved in inter-subunit interactions. The apical domains contribute nine residues for the inter-subunit interactions, while the intermediate domains contribute six residues. Overall, mHsp60 has a much smaller inter-subunit interface than GroEL, ~ 1258 Å^2^ versus ~ 1674 Å^2^. In the following, we compare the molecular interactions on the inter-subunit interface between mHsp60 and GroEL. We focus on the equatorial domains because they contribute most of the inter-subunit interactions and their regional maps are well resolved with clear sidechain information.

Center on the inter-subunit interface is a four-stranded β-sheet: β1 and β18 from one subunit connected with β2 and β3 from the other subunit (Fig. 3a). β strands are shorter in mHsp60 than in GroEL: 12 mHsp60 residues compared with 19 GroEL residues are involved in the β-sheet (Table 3). Specifically, β1 and β18 are shortened to three residues, the minimal length for β-strands, from five and seven residues in GroEL, respectively. Together, four hydrogen bonds from the backbones are lost in the reduced-size β-sheet of mHsp60 (more below).

**Table 3:** Comparison of inter-subunit interactions between mHsp60 and GroEL.

Contacts	mHsp60	GroEL
Outer side^a^		
Inner side^a^		
β-sheet^b^		

^a^Charge-charge interactions between sidechain atoms. Distances shorter than 5 Å in the GroEL structure (cryoEM structure, pdb: 5w0s) and their corresponding values in the mHsp60 structure are listed. Shade residues are from a neighboring subunit. Residues that are different between mHsp60 and GroEL are underlined. Numbers are distances in angstrom. Dashed lines are missing interactions. The K2-E522 dotted line denotes a distance longer than 5 Å, and the R36-M114 dotted line denotes a charge-polar interaction.

^b^Hydrogen bonds between backbone atoms. Arrows indicate the directions of β strands.

**Figure 4:**
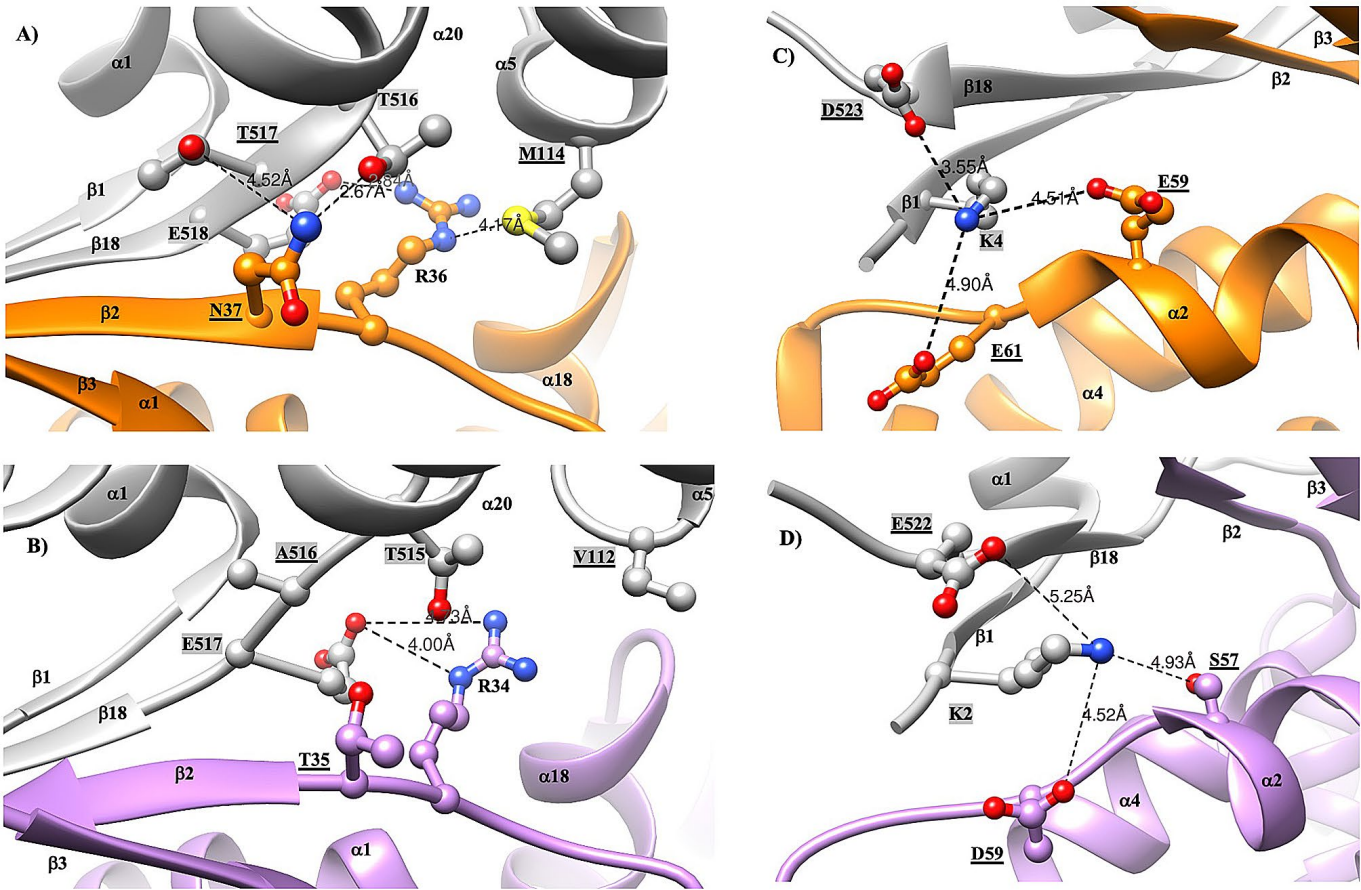
mHsp60 has reduced inter-subunit interactions compared with GroEL. Charge-charge or charge-polar interactions at the outer side (**A**, **B**) and inner side (**C**, **D**) of the β-sheet**.** mHsp60 subunits are in purple and gray, while GroEL subunits are in orange and gray. Shade residues are from a neighboring subunit; residues that are different between mHsp60 and GroEL are underlined. Numbers, distances in angstrom, are summarized in Table 3.

The shortened β strands in mHsp60 are associated with the unique mHsp60 sequences flanking the N- or C-termini of the β strands. At the outer side of the β-sheet, the GroEL N37 interacts closely with T516 (2.7 Å) and is proximity with T517 (4.5 Å); both T516 and T517 are from the other GroEL subunit (Table 3). These interactions impose restrains on the backbone of N37, leading to a twisted N-terminus of β2 at N37 (Fig. 4A). Also, they together with the R36-E518 interaction (next) result in a tight twist N-terminus of β18. The GroEL R36 forms a strong charge-charge interaction with E518 (2.8 Å) and a weak charge-polar interaction with M114 (4.2 Å) from the neighboring subunit. These restrains on T517 (via N37) and E518 (via R36) explain the T517-E518 segment tilts ~ 45° from the rest of β18 (Fig. 4A). Together, these inter-subunit sidechain interactions are important in forming the twisted conformation of the two interacting inter-subunit strands β2 and β18. However, most of inter-subunit sidechain interactions are lost in mHsp60 (Fig. 4B). The absence of T35 interactions with A516 and T515 (Table 3) releases the restrains on T35 and A516 from forming twisted N-termini of β2 and β18. Similarly, the much-reduced R34-E517 interactions (4.0/4.7 Å) and the absence of R34-V112 interaction release the restrain on E517 from a tight bent of β18. Thus, the three mHsp60 unique residues, T35, A516 and V112, diminish the restrains required to form the twisted conformations found in GroEL β2 and β18, leading to shortened β2 and β18 in mHsp60. Importantly, the three residues are conserved in higher eukaryotic chaperonins (Fig. 3c).

At the inner side of the inter-subunit β-sheet, the GroEL K4 sits snugly in the center of a negative cluster. K4 interactions with both E59 and E61 from the neighboring subunit, albeit modest, position K4 to form an effective interaction with D523 (3.6 Å). The K4-D523 sidechain interaction brings the backbones of K4 and D523 close to form hydrogen bonding, adding twisted ends to β1 and β18, respectively (Fig. 4C). In comparison, with the absence of mHsp60 K2-E522 sidechain interaction, likely affected by the different K2-S57 and K2-D59 interactions when compared with those of K4-E59 and K4-E61 (Table 3), the backbones of K2 and E522 are too far to engage in hydrogen bonding, excluding both residues from β1 and β18, respectively (Fig. 4D). Thus, the three mHsp60 residues, S57, D59 and E522, contribute to the shortened β1 and β18. Importantly, the three residues are conserved in higher eukaryotic chaperonins (Fig. 3c), as found with T35, A516 and V112 (above).

### Unfolding free energy

The above structural analysis indicates a less stable mHsp60 heptamer than a GroEL heptamer. Consistently, we found that the unfolding free energy (ΔG_unfolding_) of mHsp60 is lower than GroEL^SR^ (Table 4, Fig. 3d). Our structural analysis also shows the importance of the regions including the N-terminus in the inter-subunit interactions. D3G mutation, associated with MitCHAP60 disease^32^, has been found to destabilize the mHsp60 heptamer^33^. D3 in mHsp60 is the first residue in the already minimal three-residue β1. Since Gly has low propensity to form a β strand, D3G mutation most likely demolishes β1, downgrading the four-stranded to a three-stranded, β18-β2-β3, β-sheet. Consistently, we found that ΔG_unfolding_ of mHsp60^D3G^ was much smaller than the wildtype mHsp60 (Table 4, Fig. 3d).

**Table 4:** Thermodynamic parameters from thermal unfolding of mHsp60 and GroEL^SR^ proteins via circular dichoism.

Proteins	ΔH_vh_ (kcal/mol)^a^	ΔG_unfolding_ (kcal/mol)^b^	T_m_^c^ (°C)
mHsp60	13.92	9.59	64.7
GroEL^SR^	15.15	10.44	64.7
mHsp60^D3G^	3.49	2.36	62.8

^a^van’t Hoff enthalpy, derived from fitting data to Eq. 1.

^b^The free energy of unfolding at 20 °C, derived from Eq. 2.

^c^The temperature of the transition midpoint, derived from fitting data to Eq. 1.

### Enhanced subunit interactions in mHsp60 heptamer in response to mHsp10 binding

**Figure 5:**
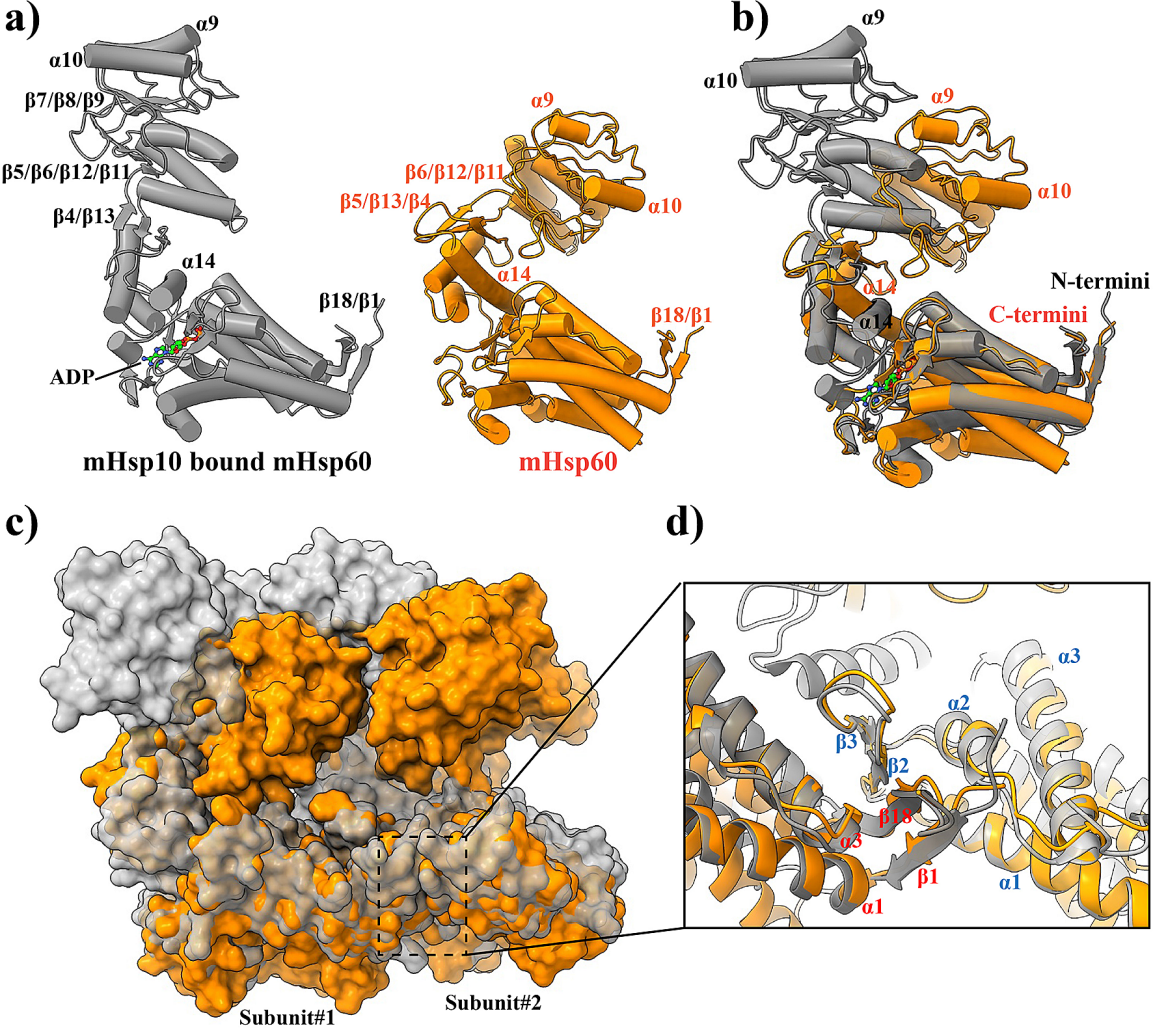
mHsp10 binding stabilizes mHsp60 subunit association. (**a**) Ribbon diagrams of mHsp60-mHsp10 (PDB: 6MRD, grey) and mHsp60 (orange) subunits. Structural elements mentioned in text are annotated. (**b**) Overlay of one mHsp60-mHsp10 subunit and one mHsp60 subunit in ribbon diagram representation. (**c**) Overlay of mHsp60 with mHsp60-mHsp10 in surface representation. The mHsp60 heptameric ring in mHsp60-mHsp10 was superimposed with the apo mHsp60 heptamer. Only two subunits from each structure are shown. (**d**) Closeup on the inter-subunit interface around the β1-β18-β2-β3 sheet. The sheet acts as a pivotal point to bring the neighboring equatorial domains closer in mHsp60-mHsp10 than in mHsp60. Secondary structures are labeled in blue for one subunit and in red for the other subunit.

The mHsp60 subunit interactions are enhanced upon mHsp10 binding. We compared our structure of single-ring mHsp60 (3.4 Å) with the structure of single-ring mHsp60-mHsp10-ADP^26^ (3.8 Å, PDB: 6MRD), both solved via cryo-EM (Fig. 5a,b). We first focused on the inter-subunit interface in the equatorial domains, since the domains provide the major inter-subunit interactions (above). Strikingly, mHsp10 binding induces significant structural changes in the equatorial domain, promoting the subunit association. The shortened the inter-subunit β1-β18-β2-β3 sheet is largely restored to that found in GroEL: β1 restores from three to five residues, while β18 increases from three to five resides (Fig. S4). Moreover, the inter-subunit β sheet serves as a pivoting point to tilt the neighboring equatorial domains towards each other (Fig. 5c,d). As a result, the interface formed via the equatorial domains increases significantly by ~ 29% from 938 Å^2^ in the mHsp60 alone structure to 1211 Å^2^ in the mHsp60-mHsp10 structure. In addition, both the apical and the intermediate domains undergo large domain movements (below), increasing their contribution to the inter-subunit interaction from 25 to 35%. Overall, the inter-subunit interface of the mHsp60 heptamer increases drastically by ~ 49% from 1258 Å^2^ in the mHsp60 alone structure to 1871 Å^2^ in the mHsp60-mHsp10 structure. The mHsp10-induced drastic increase in inter-subunit interface in mHsp60 heptamer is consistent with the observation that the presence of mHsp10 (and ATP) prevents mHsp60 from dissociation^20^.

Similarly as seen in GroEL upon GroES binding^34^, both the apical and the intermediate domains of mHsp60 undergo large domain movements upon mHsp10 binding (Fig. 5a,b). Specifically, α14 of the intermediate domain rotates downwards to the equatorial domain in mHsp60-mHsp10, closing the nucleotide binding site and locking the bound nucleotide. The three stranded β4- β13-β5 sheet in apo mHsp60 is tilted upward and reduced to a two stranded β4-β13 sheet in mHsp60-mHsp10. The connected three-stranded β6-β12-β11 sheet in mHsp60 is swung ~ 150° and increased to a four-stranded β5-β6-β12-β11 sheet in mHsp60-mHsp10. α9 and α10, the main binding site for the substrate proteins^35,36^, originally facing the chaperonin cavity in mHsp60, are repositioned upward to interact with mHsp10 in mHsp60-mHsp10. Importantly, the re-positioned apical and intermediate domains are in close contact with their counterparts in the neighboring subunits. As a result, their combined inter-subunit interface increases by > 100% from 320 Å^2^ in the mHsp60 alone structure to 660 Å^2^ in the mHsp60-mHsp10 structure, contributing to stabilizing the mHsp60 heptamer.

## Discussion

Due to its essential role for mitochondrial function and its important roles in human health, mHsp60 has been proposed as potential biomarkers and drug targets^5–8^. However, detailed molecular and structural investigations on mHsp60 mechanism have been hampered by the difficulties in purifying mHsp60 due to its intrinsic dynamics. The intrinsic dynamics, although a hurdle to detailed molecular investigations on mHsp60, has been implicated in the unique mechanism of mHsp60. Structures of mHsp60 in complex with mHsp10 have been reported^25,26^, however, these structural studies do not address the structural dynamics of mHsp60, because the mHsp60 subunit association is stabilized in the mHsp60-mHsp10 complex. Here we report the structure of mHsp60 alone, the initial functional state in the mHsp60-mHsp10 chaperone reaction. Our structural analysis provides explanations for the dynamics of the mHsp60 subunit associations and how the mHsp60 subunit association is enhanced upon mHsp10 binding.

The dynamics of mHsp60 subunit associations is due to the unique sequences at the inter-subunit interface. Many of the six unique mHsp60 sequences result in diminishing the inter-subunit sidechain interactions as found in GroEL. For example, the mHsp60 T35 does not interact with either T515 or A516 of the neighboring subunit, while the corresponding GroEL N37 interacts with T516 and T517 of the neighboring subunit. Moreover, since these unique sequences are located at the ends of β strands, losses of their sidechain interactions fail to engage their backbones to conforming to hydrogen bonding, excluding them from β strand conformation and shortening the β strands. The losses of T35 interactions with T516 and T517 exclude T35 from β2 and T516 and T517 from β18. The importance of sidechain interactions in extending β strands is underscored in GroEL. The N37 interactions with T516 and T517, together with the R36 interaction with E518, restrict the backbones of N37, T517 and E518. As a result, N37 adds a twisted N-terminus to β2, and both T517 and E518 extend a bended N-terminal segment to β18. The reduced inter-subunit sidechain interactions due to the unique mHsp60 sequences, together with the resulting shortened inter-subunit β-sheets, decrease the inter-subunit interface of mHsp60, accounting for the structural dynamics of mHsp60 subunit association. Importantly, the unique sequences, T35, S57, D59, V112, A516, and E522, are specific to and conserved within the mitochondrial chaperonins of higher eukaryotes including human. For the lower eukaryotic organisms such as yeast, the sequences at these positions resemble those of GroEL. Moreover, yeast contain sequences for one of the two conserved inter-ring salt bridges, R452-E462 in GroEL, while the higher eukaryotes lack sequences for both salt bridges (Fig. 3c). Thus, the destabilizing sequences are unique to the single ring eukaryotic chaperonins, underscoring their importance in structural dynamics and functional mechanism of the single-ring chaperonins in higher eukaryotic mitochondria.

Binding of cochaperonins induces large structural changes in chaperonins in a nucleotide dependent manner. The large structural changes include an upward rotation of α14 in the equatorial domain, an upward tilt of the β5/β13/β4 sheet in the intermediate domain, and a large swing of the β6/β12/β11 sheet and a reorientation of α9 and α10 in the apical domain (Fig. 5a). For GroEL, the large structural changes do not affect the inter-subunit interface: the interface remains largely the same before (1583 Å^2^) and after (1594 Å^2^) GroES binding (PDB: 1PCQ; the trans and cis GroEL ring, respectively). In addition, the contributions from individual domains to the inter-subunit interface remain largely the same. For example, the equatorial domains contribute 62% and 64% of the total interface before and after GroES binding, respectively. Moreover, structure of the inter-subunit β1-β18-β2-β3 sheet remains the same after GroES binding. This structural analysis suggests that the subunit association in the GroEL ring, i.e., the stability of the GroEL ring, is not affected by GroES binding. In a striking contrast, mHsp10 binding affects inter-subunit interaction in mHsp60 drastically. Upon mHsp10 binding, the overall inter-subunit interface in mHsp60 increases by 49%, and the contributions from the equatorial domains and the apical/intermediate domains increase by 29% and 105%, respectively. In addition, the shortened inter-subunit β1-β18-β2-β3 sheet of mHsp60 is restored to the same as in GroEL or the GroEL-GroES complex (Fig. S4). Thus, unlike GroEL, mHsp60 subunit association, i.e., mHsp60 heptameric stability, is enhanced in the presence of bound mHsp10. The mHsp10 effect on mHsp60 stability implicates the importance of structural dynamics in heptameric mHsp60, underscoring the mechanistic differences between mHsp60-mHsp10 and GroEL-GroES.

## Methods

### Protein purification

Gene encoding the mature mHsp60 sequence (without the mitochondrial targeting signal) was synthesized with codons optimized for protein expression in *E. coli*, and was inserted into pBbE5a with NdeI/BamHI sites. pBbE5a-mHsp60 plasmid was transformed to BL21DE3 for protein expression. Cells were grown in LB at 37 °C until OD_600_ = 0.6, protein expression was induced with 0.4 mM IPTG, and cells continued to grow for 5 h. Protein purification was modified from a published protocol^22^. Briefly, cells were suspended in Buffer A (10 mM KHEPES, pH 7.4, 1 mM EDTA, 1 mM DDT and 5% glycerol) and lyzed via sonication. After centrifugation, ammoniate sulfate was added to the clear lysate to a final concentration of 20%. The solution was centrifuged, and the supernatant was loaded onto a butyl column, preequilibrated with 20% (NH_4_)_2_SO_4_ ammoniate sulfate in Buffer A. After extensive wash including with 20% methanol, 20% ammoniate sulfate in Buffer A, the bound protein was eluted with a linear gradient of 20–0% (NH_4_)_2_SO_4_. The mHsp60 containing fractions were combined and (NH_4_)_2_SO_4_ was added to a final 60% to precipitate mHsp60. The precipitated mHsp60 was dissolved in 100 mM NaCl in Buffer A, and the solution was subjected to gel filtration purification using a Superdex 200 column (GE Healthcare). Fractions containing single ring mHsp60 were concentrated to a desired concentration, and the freshly concentrated mHsp60 was frozen on the EM grids or used within the same day for analysis. mHsp60^D3G^ was generated using QuickChange (Qiagen), and purified as above. GroEL and GroEL^SR^ were purified as before^37^.

### Dynamic light scattering (DLS)

Freshly purified mHsp60, GroEL and GroEL^SR^ were subjected to DLS analysis using a Zetasizer Nano-S dynamic light scattering instrument (Malvern Instruments). Samples were in 100 mM NaCl, 10 mM KHepes, 1 mM EDTA, 1 mM DTT and 5% glycerol, and the temperature was 25 °C. DLS data were collected within few hours of purification and for the next six days. Measurements were repeated more than once for each sample.

### ATPase assay

The steady-state ATP hydrolysis rate was measured using the malachite green assay, as described in our previous work^37^. mHsp60, GroEL or GroEL^SR^ was added to TEA buffer (50 mM triethanolamine, pH 7.5, 50 mM KCl, and 20 mM MgCl_2_) to a final concentration of 0.25 uM (heptameric mHsp60 or GroEL^SR^) or 0.125 uM (tetradecameric GroEL). The final concentration of mHsp60 or GroES was 0.3 uM. The solution was incubated at 25 °C for 10 min. The hydrolysis was initiated by addition of 100 mM pH 7.0 ATP to a final concentration of 2 mM and followed every 3 min for 21 min using the malachite green assay. Measurements were repeated more than once for each sample.

### MDH folding assay

Refolded MDH was assayed by measuring its ability to convert NADH to NAD^+^, as described in our previous work^37^. MDH was unfolded in TEA buffer including 3 M GdmHCl to a final concentration of 36.7 uM (monomeric MDH). To refold MDH, 2.75 ul of unfolded MDH was diluted at 1:100 (v/v) to a final volume of 275 ul of refolding solution (at 30 °C) containing 50 mM TEA, pH 7.4, 50 mM KCl, 1 mM ATP, and 2 uM heptameric mHsp60 or GroEL^SR^ or 2 uM tetradecameric GroEL) with 4 uM or without mHsp10 or GroES. At desired time intervals, 20 ul of reaction solution was removed and mixed with 1 ml of NADH assay solution (50 mM TrisCl, pH 7.4, 10 mM DTT, 0.2 mM NADH, 1 mM ketomalonate), and absorption at 340 nm was taken to monitor the decrease of NADH. As a positive control, activity of 367 nM native MDH (monomeric concentration) was measured at the same time intervals and was taken as 100% activity. Measurements were repeated more than once for each sample.

### Circular dichoism (CD)

Protein stocks were in 100 mM NaF, 10 mM KHEPES, pH 7.4, 1 mM EDTA, 1 mM DDT and 5% glycerol. Proteins were tenfold diluted to 100 mM NaF, 10 mM KHEPES, pH 7.4, 1 mM EDTA and 1 mM TCEP. Samples were heated from 20 to 86 °C with a heating rate of 2.0 °C/min by a Jasco Peltier thermo control device. The CD signal at 222 nm was recorded by a J-715 CD spectrometer every 2 °C with a 4-s averaging time. Measurements were repeated more than once for each sample. Thermal denaturation was analyzed by fitting the data to a two-state model according to the following equation^38^:

1 $$y = \frac{{y_{N} + m_{N} T + \left( {y_{D} + m_{D} T} \right)\exp \left[ {\Delta H_{vh} /R(1/T_{m} - 1/T)} \right]}}{{1 + \exp \left[ {\Delta H_{vh} /R(1/T_{m} - 1/T)} \right]}}$$

where y is the observed CD signal at 222 nm, *y*_N_ and *y*_D_ are the derived signals for the native and denatured samples, and *m*_N_ and *m*_D_ are the derived baseline slopes for the native and denatured samples, T is the temperature, ΔH_*vh*_ is the van’ Hoff enthalpy, R is the gas constant and T_m_ is the temperature of the transition midpoint. The free energy of unfolding at specific temperature is estimated from a modified Gibbs–Helmholtz equation, assuming no heat capacity change between the denature and native conformations: ΔCp = C_p_(denatured) − C_p_(native) = 0

2 $$\Delta G\left( T \right) = \Delta H_{vh} \left( {1 - \frac{T}{{T_{m} }}} \right) - \Delta C_{p} \left[ {\left( {T_{m} - T} \right) + Tln\left( {T/T_{m} } \right)} \right].$$

### Cryo-EM data collection, image analysis, and model building

Protein sample prepared in the same batch were submitted to two facilities PSU COM and NCCAT for cryo-EM data collection. Detailed methods for cryo-EM are described in the supplementary method section. In general, approximate 4 µl of mHsp60 in ~ 9 mg/ml were applied to Quantifoil™ holey carbon grids. Vitrification was performed using Thermo Fisher Scientific (TFS) Vitrobot Mark IV under 100% humidity. Frozen-hydrated specimens were loaded into Thermo Fisher Scientific Titan Krios operated at 300 kV equipped with Gatan K3 camera. Data collection was done by using TFS EPU or Leginon^39^ with (at 30 eV) or without energy filter. Initial data quality assessment was done by RELION and Appion^40^. Dose weighted motion corrected was done by MotionCor2^41^ and CTF estimation was done by ctffind4^42^ and Gctf^43^. Particles were semi-manually picked by e2boxer.py in EMAN2^44^. Reference-free 2D classification, initial model building, 3D classification, 3D refinement, and CTF refinement were performed in RELION^45^. A total number of 182,600 particles were used to build the final 3D reconstruction at a resolution of 3.5 Å for NCCAT data and a total number of 196,060 particles were used in the final 3D reconstructions at a resolution of 3.5 Å for PSU COM data (Table 2). To combined final 3D models, the unfiltered odd and even electron density maps from both datasets were scaled based on their size and density and summed in the real space. The combined 3D model gave an estimated resolution at 3.4 Å. Cryo-EM 3D density map was rendered by UCSF ChimeraX^46^. Atomic model for mHsp60 was built using GroEL coordinate (PDB 1PCQ) as a template. Mutation and real space refinement were performed in Coot^47^ and Phenix^48^.

## Supplementary Information

Supplementary Information.

## References

[1] Cheng MY (1989). Mitochondrial heat-shock protein hsp60 is essential for assembly of proteins imported into yeast mitochondria. Nature.

[2] Prasad TK, Hack E, Hallberg RL (1990). Function of the maize mitochondrial chaperonin hsp60: Specific association between hsp60 and newly synthesized F1-ATPase alpha subunits. Mol. Cell Biol..

[3] Koll H (1992). Antifolding activity of hsp60 couples protein import into the mitochondrial matrix with export to the intermembrane space. Cell.

[4] Ostermann J, Horwich AL, Neupert W, Hartl FU (1989). Protein folding in mitochondria requires complex formation with hsp60 and ATP hydrolysis. Nature.

[5] Nakamura H, Minegishi H (2013). HSP60 as a drug target. Curr. Pharm. Des..

[6] Pace A (2013). Hsp60, a novel target for antitumor therapy: Structure-function features and prospective drugs design. Curr. Pharm. Des..

[7] Cappello F (2014). Hsp60 chaperonopathies and chaperonotherapy: Targets and agents. Expert Opin. Ther. Targets.

[8] Meng Q, Li BX, Xiao X (2018). Toward developing chemical modulators of Hsp60 as potential therapeutics. Front. Mol. Biosci..

[9] Gupta, R. S. in *The Chaperonins* (ed R. Ellis) Ch. 2, 27–57 (Academic Press, 1996).

[10] Sigler PB (1998). Structure and function in GroEL-mediated protein folding. Annu. Rev. Biochem..

[11] Thirumalai D, Lorimer GH (2001). Chaperonin-mediated protein folding. Annu. Rev. Biophys. Biomol. Struct..

[12] Hartl FU, Hayer-Hartl M (2002). Molecular chaperones in the cytosol: From nascent chain to folded protein. Science.

[13] Horwich AL, Farr GW, Fenton WA (2006). GroEL-GroES-mediated protein folding. Chem. Rev..

[14] Lin Z, Rye HS (2006). GroEL-mediated protein folding: Making the impossible, possible. Crit. Rev. Biochem. Mol. Biol..

[15] Weber F, Keppel F, Georgopoulos C, Hayer-Hartl MK, Hartl FU (1998). The oligomeric structure of GroEL/GroES is required for biologically significant chaperonin function in protein folding. Nat. Struct. Biol..

[16] Weissman JS (1995). Mechanism of GroEL action: Productive release of polypeptide from a sequestered position under GroES. Cell.

[17] Viitanen PV (1992). Mammalian mitochondrial chaperonin 60 functions as a single toroidal ring. J. Biol. Chem..

[18] Brocchieri L, Karlin S (2000). Conservation among HSP60 sequences in relation to structure, function, and evolution. Protein Sci..

[19] Nielsen KL, Cowan NJ (1998). A single ring is sufficient for productive chaperonin-mediated folding in vivo. Mol. Cell.

[20] Levy-Rimler G (2001). The effect of nucleotides and mitochondrial chaperonin 10 on the structure and chaperone activity of mitochondrial chaperonin 60. Eur. J. Biochem..

[21] Enriquez AS (2017). The human mitochondrial Hsp60 in the APO conformation forms a stable tetradecameric complex. Cell Cycle.

[22] Ishida R (2018). Physicochemical properties of the mammalian molecular chaperone HSP60. Int. J. Mol. Sci..

[23] Vilasi S (2017). Chaperonin of group I: Oligomeric spectrum and biochemical and biological implications. Front. Mol. Biosci..

[24] Parnas A (2012). Identification of elements that dictate the specificity of mitochondrial Hsp60 for its co-chaperonin. PLoS ONE.

[25] Nisemblat S, Yaniv O, Parnas A, Frolow F, Azem A (2015). Crystal structure of the human mitochondrial chaperonin symmetrical football complex. Proc. Natl. Acad. Sci. U. S. A..

[26] Gomez-Llorente Y (2020). Structural basis for active single and double ring complexes in human mitochondrial Hsp60-Hsp10 chaperonin. Nat. Commun..

[27] Viitanen PV (1998). Purification of mammalian mitochondrial chaperonin 60 through in vitro reconstitution of active oligomers. Methods Enzymol..

[28] Klebl DP (2021). Cryo-EM structure of human mitochondrial HSPD1. iScience.

[29] Okamoto T (2015). Functional structure and physiological functions of mammalian wild-type HSP60. Arch. Biochem. Biophys..

[30] Braig K, Adams PD, Brunger AT (1995). Conformational variability in the refined structure of the chaperonin GroEL at 2.8 A resolution. Nat. Struct. Biol..

[31] Chaudhry C (2003). Role of the gamma-phosphate of ATP in triggering protein folding by GroEL-GroES: function, structure and energetics. EMBO J..

[32] Magen D (2008). Mitochondrial hsp60 chaperonopathy causes an autosomal-recessive neurodegenerative disorder linked to brain hypomyelination and leukodystrophy. Am. J. Hum. Genet..

[33] Parnas A (2009). The MitCHAP-60 disease is due to entropic destabilization of the human mitochondrial Hsp60 oligomer. J. Biol. Chem..

[34] Xu Z, Horwich AL, Sigler PB (1997). The crystal structure of the asymmetric GroEL-GroES-(ADP)7 chaperonin complex. Nature.

[35] Fenton WA, Kashi Y, Furtak K, Horwich AL (1994). Residues in chaperonin GroEL required for polypeptide binding and release. Nature.

[36] Chen L, Sigler PB (1999). The crystal structure of a GroEL/peptide complex: Plasticity as a basis for substrate diversity. Cell.

[37] Illingworth M, Ramsey A, Zheng Z, Chen L (2011). Stimulating the substrate folding activity of a single ring GroEL variant by modulating the cochaperonin GroES. J. Biol. Chem..

[38] Santoro MM, Bolen DW (1988). Unfolding free energy changes determined by the linear extrapolation method. 1. Unfolding of phenylmethanesulfonyl alpha-chymotrypsin using different denaturants. Biochemistry.

[39] Suloway C (2005). Automated molecular microscopy: The new Leginon system. J. Struct. Biol..

[40] Lander GC (2009). Appion: An integrated, database-driven pipeline to facilitate EM image processing. J. Struct. Biol..

[41] Zheng SQ (2017). MotionCor2: Anisotropic correction of beam-induced motion for improved cryo-electron microscopy. Nat. Methods.

[42] Rohou A, Grigorieff N (2015). CTFFIND4: Fast and accurate defocus estimation from electron micrographs. J. Struct. Biol..

[43] Zhang K (2016). Gctf: Real-time CTF determination and correction. J. Struct. Biol..

[44] Tang G (2007). EMAN2: An extensible image processing suite for electron microscopy. J. Struct. Biol..

[45] Scheres SH (2012). RELION: Implementation of a Bayesian approach to cryo-EM structure determination. J. Struct. Biol..

[46] Goddard TD (2018). UCSF ChimeraX: Meeting modern challenges in visualization and analysis. Protein Sci..

[47] Emsley P, Cowtan K (2004). Coot: Model-building tools for molecular graphics. Acta Crystallogr. D Biol. Crystallogr..

[48] Adams PD (2010). PHENIX: A comprehensive Python-based system for macromolecular structure solution. Acta Crystallogr. D Biol. Crystallogr..

