# Publisher Correction: Structural basis for the structural dynamics of human mitochondrial chaperonin mHsp60

**DOI:** 10.1038/s41598-021-96945-w

**Published:** 2021-08-24

**Authors:** Joseph Che-Yen Wang, Lingling Chen

**Affiliations:** 1 Department of Microbiology and Immunology, The Pennsylvania State University College of Medicine, 500 University Drive, Hershey, PA 17033 USA; 2 Department of Molecular and Cellular Biochemistry, 212 S. Hawthorne Dr, Bloomington, IN 47405 USA

Correction to: *Scientific Reports* 10.1038/s41598-021-94236-y, published online 20 July 2021

In the original version of this Article Joseph Che-Yen Wang was incorrectly affiliated with ‘Department of Molecular and Cellular Biochemistry, 212 S. Hawthorne Dr., Bloomington, IN, 47405, USA’. In addition, Lingling Chen was incorrectly affiliated with ‘Department of Microbiology and Immunology, The Pennsylvania State University College of Medicine, 500 University Drive, Hershey, PA, 17033, USA’.

The correct affiliations are listed below.

Affiliation 1:

Department of Microbiology and Immunology, The Pennsylvania State University College of Medicine, 500 University Drive, Hershey, PA, 17033, USA

Joseph Che-Yen Wang

Affiliation 2:

Department of Molecular and Cellular Biochemistry, 212 S. Hawthorne Dr., Bloomington, IN, 47405, USA

Lingling Chen

The original Article has been corrected.

