# Supplementary material for: Structural basis for the structural dynamics of human mitochondrial chaperonin mHsp60
Source: Sci Rep. 2021 Jul 20;11:14809. doi: 10.1038/s41598-021-94236-y (PMC8292379; doi:10.1038/s41598-021-94236-y)
Supplement: Supplementary file 1 — Supplementary Information. [file 41598_2021_94236_MOESM1_ESM.docx]

**Supplementary Information**

**Structural basis for the structural dynamics of human mitochondrial chaperonin mHsp60**

Joseph Che-Yen Wang^1*^ and Lingling Chen^2*^

**Supplementary method:**

*Cryo-EM data collection and image analysis.* Protein sample purified and prepared in the same batch were submitted to two facilities Penn State University College of Medicine (PSU COM) and NIH National Center for CryoEM Access and Training (NCCAT) for cryo-EM data collection. For data collection at PSU COM, 4 µl of mHsp60 solution in ~9 mg/ml were applied on glow-discharged Quantifoil^TM^ R2/2 copper grids. Vitrification was performed by Thermo Fisher Scientific (TFS) Vitrobot Mark IV (blotting force = 4, blotting time = 4.5 s) at 4 ^o^ C under 100% humidity. Frozen-hydrated specimens was loaded into TFS Titan Krios operated at 300 kV equipped with Gatan BioContinuum^TM^ using K3 direct electron detector with an energy slit of 30 eV. Data collection was set up using TFS EPU software under counting mode with a total accumulated dose of 44 e^-^/Å^2^ (each frame was set to have 1 e^-^/Å^2^). A nominal magnification of 120,000x that yields a pixel size of 0.84 Å was used to collect data at a nominal defocus range of 0.8 – 3 µm. Dose weighted motion correction was done by MotionCor2 and CTF estimation was done by Gctf^2^. Both processes were performed using RELION (v3.1) software.

For NCCAT data, approximate 4 µl of mHsp60 in ~9 mg/ml were pipetted on plasma cleaned Quantifoil^TM^ UltrAuFoil^®^ R1.2/1.3 grids. Vitrification was performed by using Thermo Fisher Scientific Vitrobot Mark IV (blotting force = 0, blotting time = 3 ~ 4.5 s) at 20 ^o^ C under 100% humidity. Frozen-hydrated specimens was loaded into TFS Titan Krios operated at 300 kV equipped with Gatan K3 camera. Data collection workflow and initial data quality assessment were done by using Leginon^3^ and Appion^4^. Movie frames were collected using super-resolution mode with electron counting at a dose rate of 25 – 28 e^-^/Å^2^/s with total accumulated dose around 50 – 57 e^-^/Å^2^ and a nominal defocus range of 0.7 – 8 µm. Dose weighted motion correction was done by MotionCor2 with a binning factor of 2 that yields a calculated pixel size of 1.069 Å in the micrographs. CTF estimation was done by ctffind4^5^.

For both datasets, same data processing scheme was employed. Basically, particles were semi-manually picked by e2boxer.py in EMAN2^6^. Approximately 10,000 particles were first manually picked. Reference-free 2D classification, initial model building, and 3D refinement was performed using RELION^7^. Note that the initial model was built de novo and independently for each dataset. The low resolution 3D model was then used as a reference in e2boxer.py for automation particle picking. The particle picking result was further adjusted manually for every micrograph to ensure most particles were selected. From here, all the following data processing was done in RELION. Reference-free 2D classification were performed multiple times to remove ice-contaminated and background density. The model used for particle picking was also used for initial 3D classification and 3D refinement with C7 symmetry imposition. Data processing started with down scaled pixel size at bin=4 and gradually moved up to un-binned pixel size. Once the 3D refinement converged, CTF refinement and particle polishing were performed. A total number of 196,060 particles and a total number of 182,600 particles were used in the final 3D reconstructions for datasets collected at PSU COM and NCCAT, respectively. Both 3D reconstructions were estimated at 3.5 Å using at FSC of 0.143. The 3D density maps were rendered and analyzed by UCSF Chimera^8^ and ChimeraX^9^.

To combine both datasets, we choose to combine the final 3D reconstructions^10^. We first normalized the density of the two final maps to the same mean and standard deviation. We then calculated the optimizing scaling factor between two final maps using real-space correlation. The pixel size of one map was scaled at ± 0.0001 intervals for several steps and calculated the cross-correlation with another map. We obtained the highest correlation at 0.9834 when the scaled pixel size is at 1.0009x of the original pixel size. Note that the size difference (relative scaling factor) between these two 3D reconstructions (collected from two different microscope) was only measured at 1.0009x. Subsequently, the density and size of the even and odd 3D half maps from both dataset were normalized and scaled. Two odd half maps and two even half maps were combined in the real space, respectively. Finally, post-processing was performed for the combined unfiltered odd and even reconstructions to yield the final B-factor corrected 3D reconstruction at 3.4 Å.

**Supplementary figures:**

**
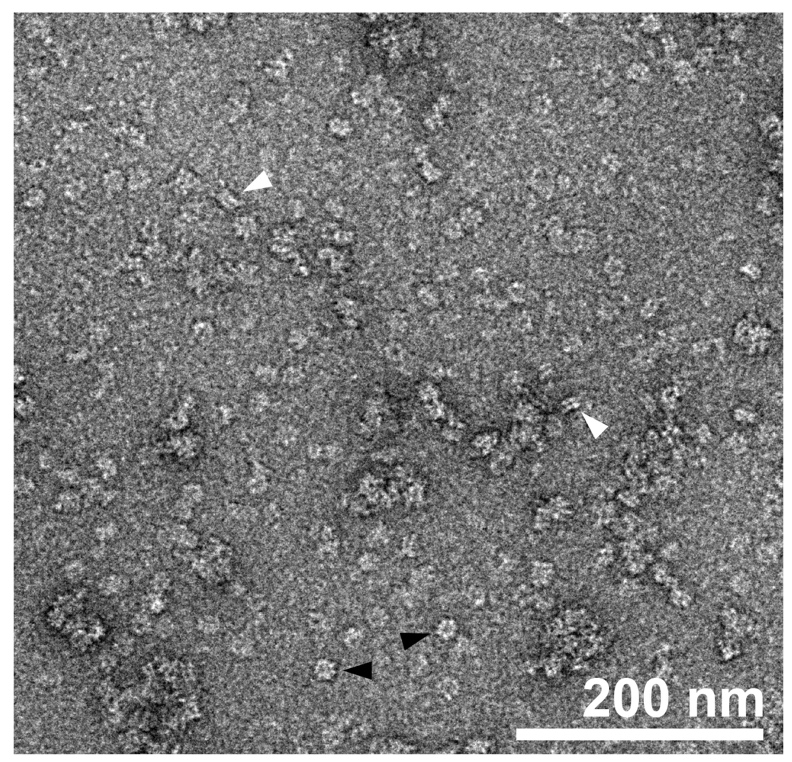
**

**Figure S1:** **mHsp60 is in single-ring conformation.** Representative micrograph of negative stained TEM. Side and tilt views are highlighted by white and black arrows.


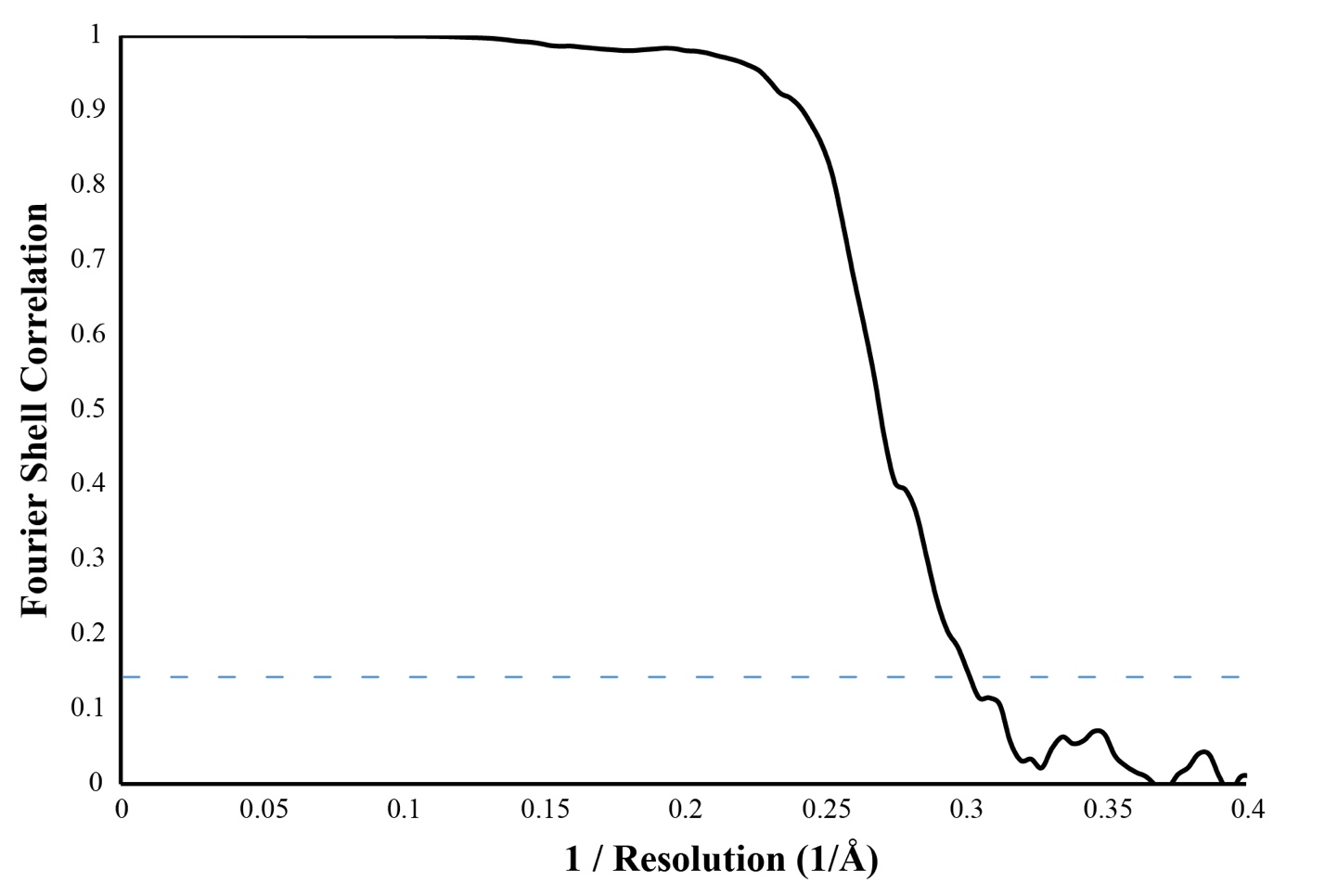


**Figure S2:** **Resolution estimation.**  Resolution was estimated using gold-standard method implemented in RELION 3.1 where the Fourier shell correlation first intersects with 0.143 (light blue dashed line) was defined as the resolution for the structure.


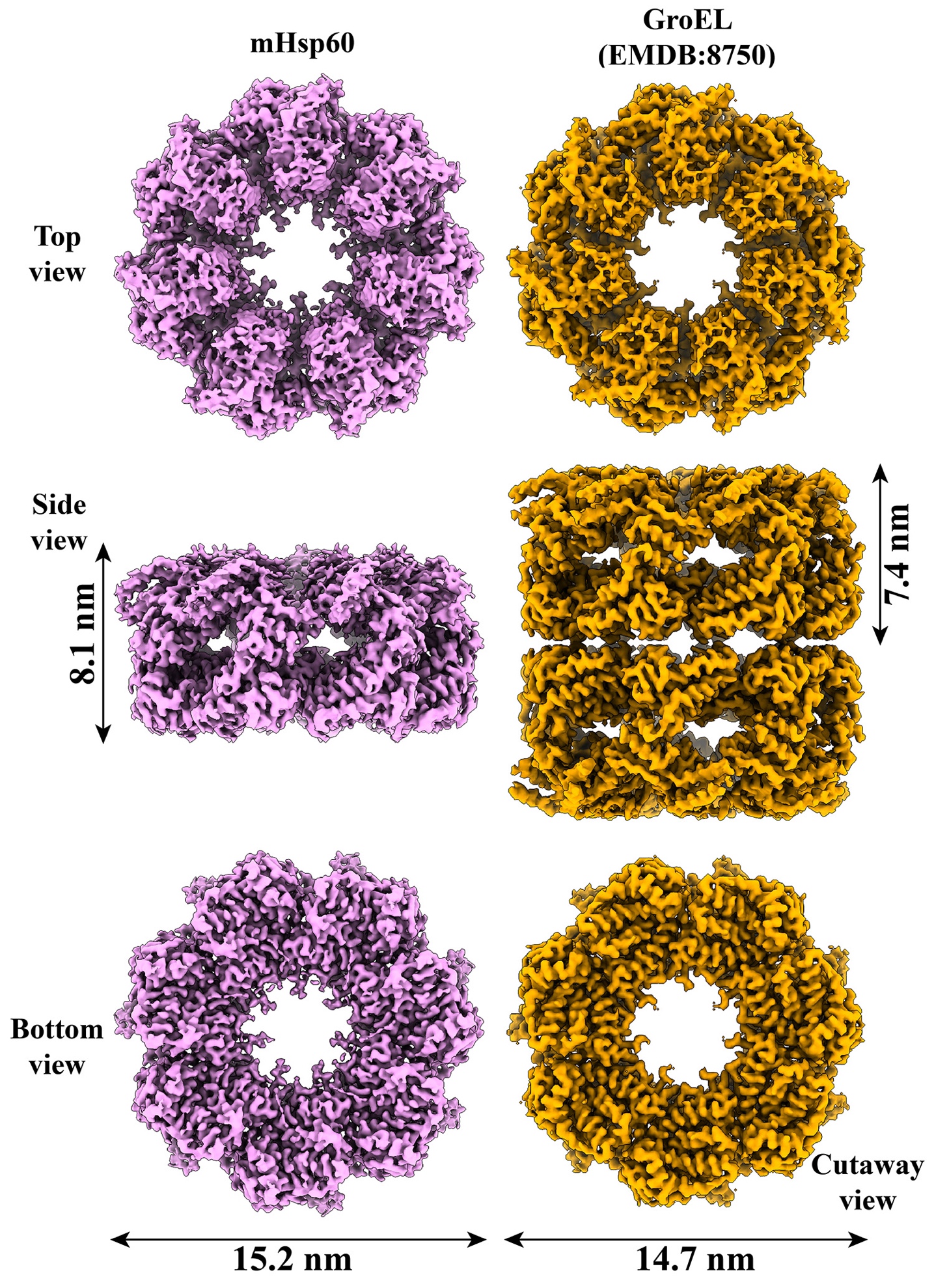


**Figure S3:** **Cryo-EM structural comparisons between mHsp60 and GroEL.** mHsp60 cryo-EM structure was compared to the published GroEL structure^1^ (EMDB: 8750). The GroEL structure at the resolution of 3.5 Å exhibits back-to-back double-ring morphology. Different to GroEL, mHsp60 contains only 7 subunits in a single ring conformation. From the top view, the apical domain appears to be more expanded in mHsp60. This region also involves in co-chaperonin binding. The apical domain is structurally more ordered in the double-ring conformation as observed in the electron density. From the side view, mHsp60 shares approximately half height of the GroEL. To compare the bottom view, one heptameric ring was segmented and removed from GroEL structure (bottom panel, right). Overall, the dimension of mHsp60 is larger than that of GroEL.

mHsp60

mHsp60-mHsp10

GroEL

GroEL-GroES

mHsp60

mHsp60-mHsp10

GroEL

GroEL-GroES

mHsp60

mHsp60-mHsp10

GroEL

GroEL-GroES

mHsp60

mHsp60-mHsp10

GroEL

GroEL-GroES

mHsp60

mHsp60-mHsp10

GroEL

GroEL-GroES

mHsp60

mHsp60-mHsp10

GroEL

GroEL-GroES

mHsp60

--AKDVKFGADARALMLQGVDLLADAVAVTMGPKGRTVIIEQSWGSPKVTKDGVTVAKSI 58

mHsp60-m

GroEL

MAAKDVKFGNDARVKMLRGVNVLADAVKVTLGPKGRNVVLDKSFGAPTITKDGVSVAREI 60

α1

α2

β1

β2

β3

human DLKDKYKNIGAKLVQDVANNTNEEAGDGTTTATVLARSIAKEGFEKISKGANPVEIRRGV 118

GroEL ELEDKFENMGAQMVKEVASKANDAAGDGTTTATVLAQAIITEGLKAVAAGMNPMDLKRGI 120

α3

α4

α5
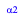


human MLAVDAVIAELKKQSKPVTTPEEIAQVATISANGDKEIGNIISDAMKKVGRKGVITVKDG 178

GroEL DKAVTAAVEELKALSVPCSDSKAIAQVGTISANSDETVGKLIAEAMDKVGKEGVITVEDG 180

α5

α6

α7

β4

human KTLNDELEIIEGMKFDRGYISPYFINTSKGQKCEFQDAYVLLSEKKISSIQSIVPALEIA 238

GroEL TGLQDELDVVEGMQFDRGYLSPYFINKPETGAVELESPFILLADKKISNIREMLPVLEAV 240

α9

α8

β6

β5

β7

β8

β9

human NAHRKPLVIIAEDVDGEALSTLVLNRLKVGLQVVAVKAPGFGDNRKNQLKDMAIATGGAV 298

GroEL AKAGKPLLIIAEDVEGEALATLVVNTMRGIVKVAAVKAPGFGDRRKAMLQDIATLTGGTV 300

α9'

α10

α11

β10

β11

human FGEEGLTLNLEDVQPHDLGKVGEVIVTKDDAMLLKGKGDKAQIEKRIQEIIEQLDVTT-S 357

GroEL ISEE-IGMELEKATLEDLGQAKRVVINKDTTTIIDGVGEEAAIQGRVAQIRQQIEEAT-S 358

α12

α13

β12

β13

α14

mHsp60

mHsp60-mHsp10

GroEL

GroEL-GroES

mHsp60

mHsp60-mHsp10

GroEL

GroEL-GroES

mHsp60

mHsp60-mHsp10

GroEL

GroEL-GroES

human EYEKEKLNERLAKLSDGVAVLKVGGTSDVEVNEKKDRVTDALNATRAAVEEGIVLGGGCA 417

GroEL DYDREKLQERVAKLAGGVAVIKVGAATEVEMKEKKARVEDALHATRAAVEEGVVAGGGVA 418

α15

β14

α16

β15

α17

human LLRCIPALDSLTPANEDQKIGIEIIKRTLKIPAMTIAKNAGVEGSLIVEKIMQSS---SE 474

GroEL LIRVASKLADLRGQNEDQNVGIKVALRAMEAPLRQIVLNCGEEPSVVANTVK---GGDGN 475

α17

α18 α18'

α19

human VGYDAMAGDFVNMVEKGIIDPTKVVRTALLDAAGVASLLTTAEVVVTEIPKEEKD-PGMG 533

GroEL YGYNAATEEYGNMIDMGILDPTKVTRSALQYAASVAGLMITTECMVTDLPKNDAADLGAA 535

α20

β17

β18

β19

β16

**Figure S4: mHsp10 binding increases the secondary structure content of mHsp60.** PDB codes: 6MRD for mHsp60-mHsp10, and 1PCQ for GroEL and GroEL-GroES. GroEL and GroEL-GroES refer to the trans and cis GroEL ring in GroEL-GroES complex, respectively. See Fig. 3C for other annotations.

**References:**

1 Roh, S. H. *et al.* Subunit conformational variation within individual GroEL oligomers resolved by Cryo-EM. *P Natl Acad Sci USA* **114**, 8259-8264, doi:10.1073/pnas.1704725114 (2017).

2 Zhang, K. Gctf: Real-time CTF determination and correction. *J Struct Biol* **193**, 1-12, doi:10.1016/j.jsb.2015.11.003 (2016).

3 Suloway, C. *et al.* Automated molecular microscopy: the new Leginon system. *J Struct Biol* **151**, 41-60, doi:10.1016/j.jsb.2005.03.010 (2005).

4 Lander, G. C. *et al.* Appion: an integrated, database-driven pipeline to facilitate EM image processing. *J Struct Biol* **166**, 95-102, doi:10.1016/j.jsb.2009.01.002 (2009).

5 Rohou, A. & Grigorieff, N. CTFFIND4: Fast and accurate defocus estimation from electron micrographs. *J Struct Biol* **192**, 216-221, doi:10.1016/j.jsb.2015.08.008 (2015).

6 Tang, G. *et al.* EMAN2: an extensible image processing suite for electron microscopy. *J Struct Biol* **157**, 38-46, doi:10.1016/j.jsb.2006.05.009 (2007).

7 Scheres, S. H. RELION: implementation of a Bayesian approach to cryo-EM structure determination. *J Struct Biol* **180**, 519-530, doi:10.1016/j.jsb.2012.09.006 (2012).

8 Goddard, T. D., Huang, C. C. & Ferrin, T. E. Visualizing density maps with UCSF Chimera. *J Struct Biol* **157**, 281-287, doi:10.1016/j.jsb.2006.06.010 (2007).

9 Goddard, T. D. *et al.* UCSF ChimeraX: Meeting modern challenges in visualization and analysis. *Protein Sci* **27**, 14-25, doi:10.1002/pro.3235 (2018).

10 Wilkinson, M. E., Kumar, A. & Casanal, A. Methods for merging data sets in electron cryo-microscopy. *Acta Crystallogr D Struct Biol* **75**, 782-791, doi:10.1107/S2059798319010519 (2019).
